# Are Anatomical Gift Donors Demographically Representative of the American Aging Patient Population?

**DOI:** 10.1093/geroni/igab046.2379

**Published:** 2021-12-17

**Authors:** Amanda Collins, Yasmin Carter

**Affiliations:** 1 University of Massachusetts Boston, Boston, Massachusetts, United States; 2 University of Massachusetts Medical School, Worcester, Massachusetts, United States

## Abstract

Body donation for medical education is voluntary and open to all; however, it is undetermined if the donors studied at UMass Medical School (UMMS) are demographically representative of the national patient population. If not, medical students are missing the opportunity of experiencing normal variation within the population, which may promote bias in their clinical years. This cross-sectional study compared data from the UMMS Anatomical Gift Program (AGP) with the Health and Retirement Study (HRS) population data. This study examined sex, race, ethnicity, veteran status, and sexual orientation. 5 years (n=540) of AGP data and 3 waves (n=5,037) of HRS data were examined. The results demonstrate that sex differences between the AGP and HRS populations (55% for females vs. 45% for males; p=.10) are NOT significant. A significant racial difference between populations is noted (p=.000), with 98.3% of the AGP vs. 72.7% of the HRS identifying as white. Veterans are overrepresented in the AGP (22.6% AGP vs. 9.6% HRS; p=.000). 12.3% of HRS participants report Hispanic ethnicity compared to 0% in the AGP. In 2016, HRS included sexual orientation, with 92.7% of respondents identifying as heterosexual, 2.6% gay or lesbian, 1.0% bisexual and 1.3% other. No data were collected by the AGP pertaining to sexual orientation and neither database ask about gender identity. Aging populations are not represented in the anatomy labs at UMMS and likely nationally. Efforts are needed to improve this and enhance the education of the medical professionals, while expanding the end-of-life options for all community members.

